# A Conversation with Julie Dunne

**DOI:** 10.1021/acscentsci.3c00403

**Published:** 2023-04-11

**Authors:** Carolyn Wilke

Thousands of years ago, Bronze Age artisans made whimsical baby bottles sculpted to look like animals with round bodies, some with perky ears and others with tails doubling as spouts. But there’s more to the vessels than meets the eye. Lipid leftovers of past feedings cling to these ceramic containers. Biomolecular archeologist Julie Dunne of the University of Bristol extracts such residues from ancient pottery and along with them clues about what people ate long ago. This work differs from the archeology that focuses on those who were once high and mighty—“bling–bling archeology,” as she calls it. For her, research into the past has always been more about how ordinary people lived.

**Figure:**
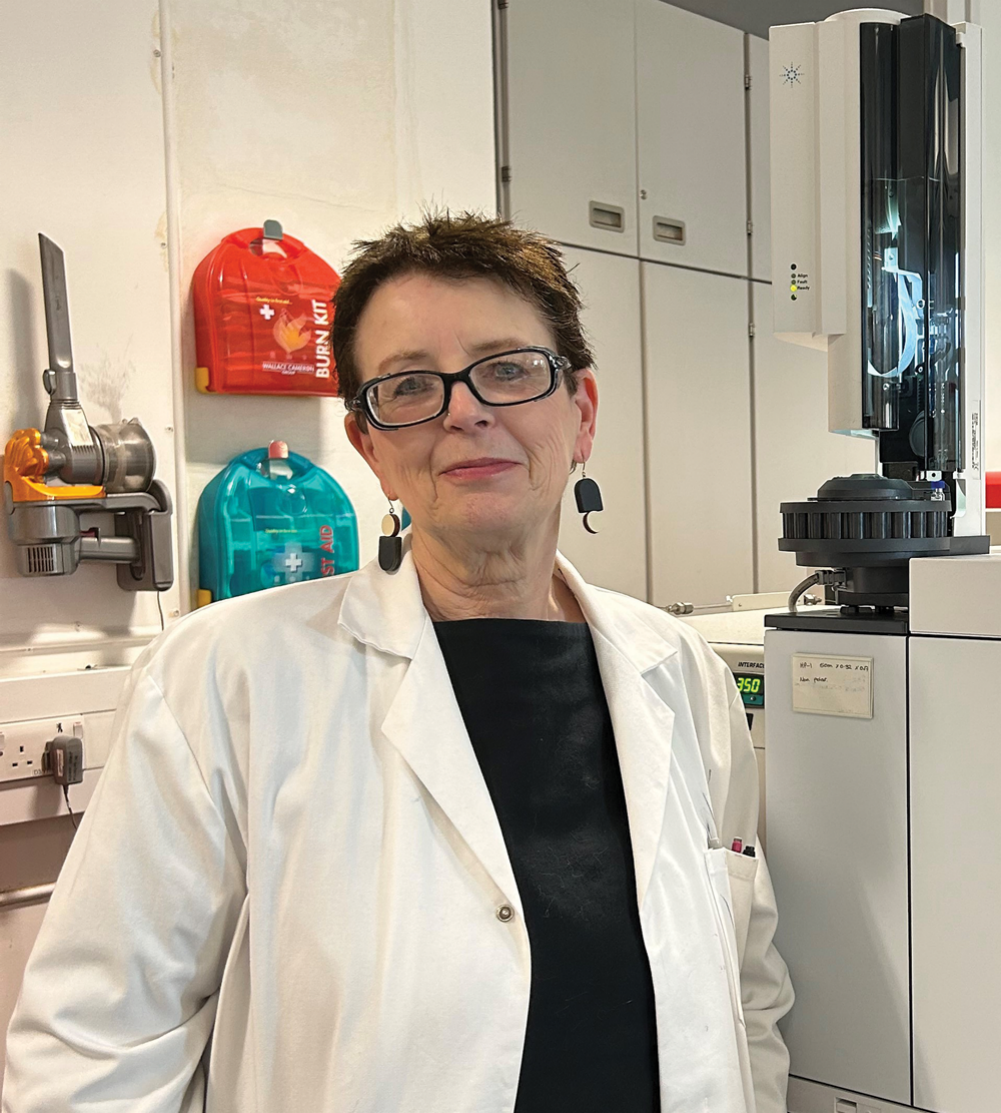
Credit: Courtesy of Julie Dunne

Her passion for unveiling hints about life long ago drove Dunne from working in accounting to pursuing a PhD and becoming an archeologist as a second career. She now collaborates with Richard Evershed, who created the field of ancient lipid analysis decades ago, and they continue to advance methods to squeeze more information from these relic biomolecules.

Because of lipids’ inherent link to food, they present a spring of information not easily obtained from ancient DNA, which typically gives more insights into population movement and species’ evolution, or from old proteins, whose analysis isn’t as robust, Dunne says. The researchers have used lipids to deduce a menu for medieval peasants, follow a beeswax trail to map prehistoric honey collecting, and track the evolution of lactase persistence. That evolved trait allows many humans to digest dairy in adulthood across Europe.

Carolyn Wilke talked with Dunne about this work. This interview was edited for length and clarity.

## Why are ancient pots a good lens through which to study the lipids humans left behind millennia ago?

Pottery dates back 17,000 or 18,000 years and is the most ubiquitous artifact found on archeological sites. Pots might look quite dense, but they have a pore structure. When people are cooking foods in their pots, lipids—the fats, oils, and waxes of the natural world—absorb into the ceramic matrix of a pot. These lipids are exactly the right size to fit in the little holes in a ceramic matrix in this amazing, serendipitous piece of fate.

The pot might have been used, say, 5,000 years ago and eventually would break and probably be discarded and buried. And then thousands of years later, some archeologists come dig it up. By that time, it’s probably just a very small piece of broken pot, and just looking at it, we don’t really know much about the past lives of people. But the lipids can tell us an awful lot about a variety of archeological questions.

## How do you collect and analyze the lipids?

We can take that very small piece of pot, grind it up, and use a set of chemical techniques to extract the lipids. We do a couple of types of extraction, but our main method is using an acid and methanol to release the lipids.

Then we use a gas chromatograph, and that essentially tells us if there were any foods processed in that pot. If it was used for cooking in the past, then the most common things we see are degraded animal fats—mainly C_16_ and C_18_ fatty acids. But they might not necessarily be all fatty acids. In the gas chromatography/mass spectrometry data, there might be beeswax, which suggests the processing of honey, or maybe resins or possibly plant material.

[As a final step], we run the samples on an isotope-ratio mass spectrometer. And that measures the stable carbon isotope values of the fatty acids to differentiate between the fat of ruminant and nonruminant animals.

**Figure:**
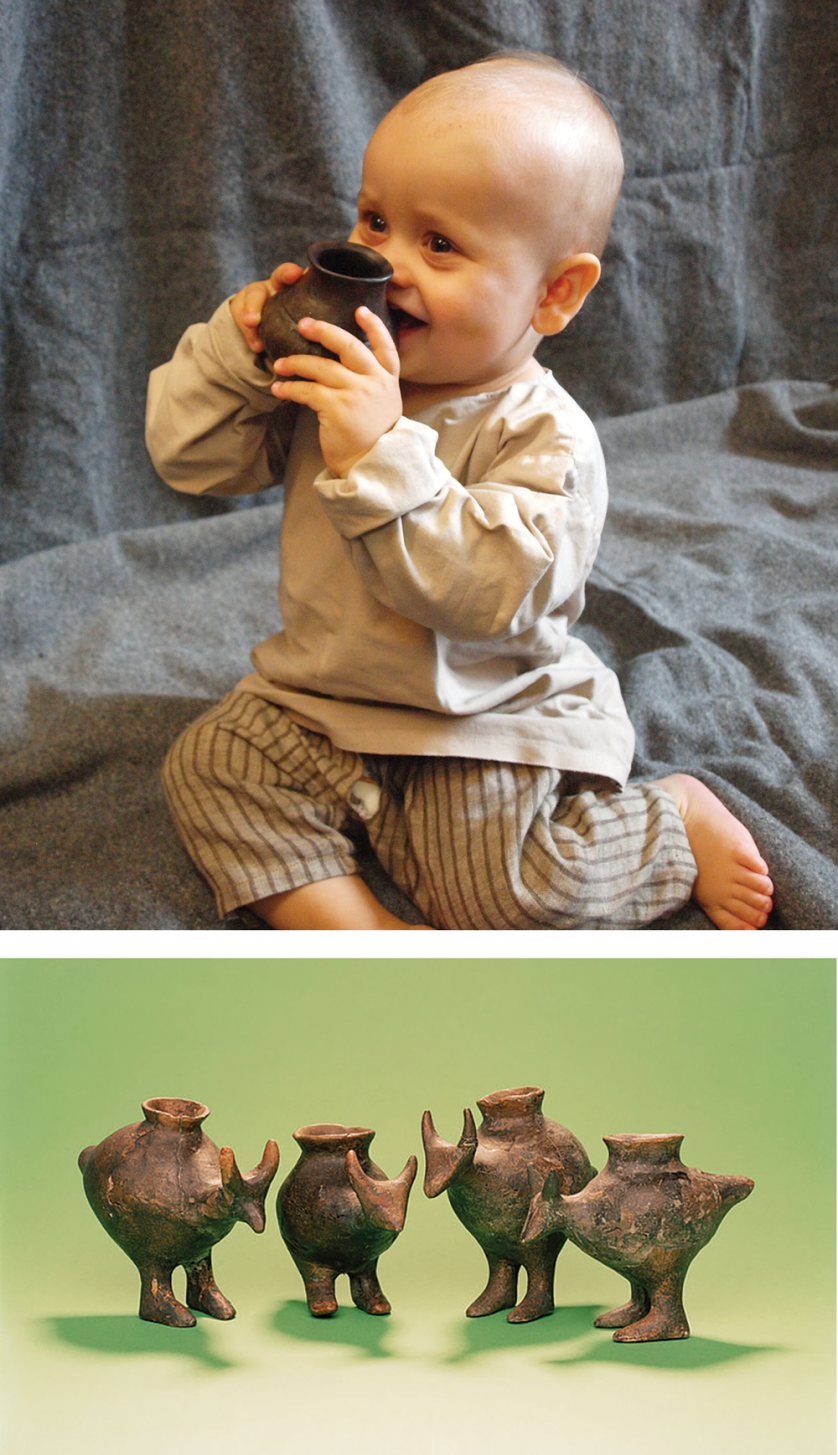
A modern-day baby using an infant-feeding vessel reconstructed from examples that date back to the Bronze Age (top). Original late Bronze Age feeding vessels from Vösendorf, Austria (bottom). Credit: Helena Seidl da Fonseca (baby)/Enver-Hirsch © Wien Museum (original vessels)

## What do lipids reveal about societies of the past?

Lipids can provide information on how humans are managing their animals. For example, in Europe, we know from the isotope values of the fatty acids and from the environment that once people domesticated animals, they were living fairly settled lifestyles. In North Africa, people domesticated animals too, but they managed them in a completely different way—they were living on plains at one part of the year and going up into the mountains in the summer, when it’s much hotter. We can see that through a combination of archeological evidence and isotope values of dairy lipids.

Lipids also tell us about when people start to exploit dairy products, an important source of fat and protein. Prior to domestication of animals, all humans were lactose tolerant when they were infants feeding from their mothers’ milk, and they were weaned once the gene [responsible for lactose tolerance] was turned off. But then we humans started to exploit animal milk, a very good example of gene-culture coevolution [in which culture influences the selection of genes]. It led to many of us becoming lactose tolerant in adulthood too. Looking at lipids in pots has allowed us to track that evolution of dairying across Europe from the Near East.

In the last 3 or 4 years, we’ve developed a technique where we can radiocarbon date specific lipids, which is a revolutionary development. So if we want to look at the evolution of dairying, we can directly date lipids and find out how old dairying’s inception was in a particular site or region.

## How does this research on past human foods connect to people today?

Looking at the lipids from feeding vessels placed in child graves enabled us to understand that people were feeding their babies with animal milk. When I did the baby bottle analysis, we really knew nothing about how people in prehistory looked after their babies. But those bottles were all unique vessels clearly designed to make babies smile, and that really brought home that parents in prehistory must have loved and cared for their children in exactly the same way that we do today.

People had time to make beautiful things. We see an ingeniousness and sense of pride in their work and their craftsmanship in making things like that. It wasn’t just a hard, bitter life where people were scrambling for food and getting by. They were the same as us, which I think is quite lovely to think about.

## Carolyn Wilke is a freelance contributor to

Chemical & Engineering News, *the independent news outlet of the American Chemical Society.*

